# Myelin Oligodendrocyte Glycoprotein Antibody-Associated Disorders: Toward a New Spectrum of Inflammatory Demyelinating CNS Disorders?

**DOI:** 10.3389/fimmu.2018.02753

**Published:** 2018-11-29

**Authors:** Franziska Di Pauli, Thomas Berger

**Affiliations:** Clinical Department of Neurology, Medical University of Innsbruck, Innsbruck, Austria

**Keywords:** multiple sclerosis, myelin oligodendrocyte glycoprotein antibody-associated disorders, neuromyelitis optica spectrum disease, inflammatory demyelinating CNS syndromes, clinically isolated syndrome

## Abstract

Inflammatory demyelinating CNS syndromes include, besides their most common entity multiple sclerosis (MS), several different diseases of either monophasic or recurrent character—including neuromyelitis optica spectrum disorders (NMOSDs) and acute disseminated encephalomyelitis (ADEM). Early diagnostic differentiation is crucial for devising individual treatment strategies. However, due to overlapping clinical and paraclinical features diagnosis at the first demyelinating event is not always possible. A multiplicity of potential biological markers that could discriminate the different diseases was studied. As the use of autoantibodies in patient management of other autoimmune diseases, is well-established and evidence for the critical involvement of B cells/antibodies in disease pathogenesis in inflammatory demyelinating CNS syndromes increases, antibodies seem to be valuable diagnostic tools. Since the detection of antibodies against aquaporin-4 (AQP-4), the understanding of immunopathogenesis and diagnostic management of NMOSDs has dramatically changed. However, for most inflammatory demyelinating CNS syndromes, a potential antigen target is still not known. A further extensively studied possible target structure is myelin oligodendrocyte glycoprotein (MOG), found at the outermost surface of myelin sheaths and oligodendrocyte membranes. With detection methods using cell-based assays with full-length, conformationally correct MOG, antibodies have been described in early studies with a subgroup of patients with ADEM. Recently, a humoral immune reaction against MOG has been found not only in monophasic diseases, but also in recurrent non-MS diseases, particularly in pediatric patients. This review presents the findings regarding MOG antibodies as potential biological markers in discriminating between these different demyelinating CNS diseases, and discusses recent developments, clinical implementations, and data on immunopathogenesis of MOG antibody-associated disorders.

## Introduction

Inflammatory demyelinating CNS diseases are a heterogeneous group, covering monophasic and multiphasic diseases, prognoses ranging from benign to fulminant, and a variety of different treatment responses. Although the sensitivity and specificity of diagnostic criteria, particularly for multiple sclerosis (MS), the most common demyelinating CNS disease, have significantly improved (1), misdiagnosis is not infrequent and occurs in up to 10% of cases (2). Differential diagnoses are beside other neurological non-inflammatory diseases, in particular neuromyelitis optica spectrum disorder (NMOSD), acute disseminated encephalomyelitis (ADEM), multiphasic disseminated encephalomyelitis (MDEM), and atypical demyelinating CNS syndromes (3, 4). Diagnosis is based on a combination of anamnesis, clinical presentation, and radiological findings (1, 5, 6) and allows, for the most part, correct stratification.

Given the recommendation for early treatment initiation in MS, and the availability of highly effective treatments (7), in the last few years efforts have been made to establish the diagnosis as early as possible. However, this in turn increases the risk of beginning a possibly harmful treatment regimen in patients without MS. The first detection of a laboratory biomarker in MS concerned the description of oligoclonal bands (OCBs) more than 60 years ago (8). However, so far analysis of the target antigen of an intrathecal immunoreaction has not been successful, and no specific antibodies have been found to be associated with MS (9).

In 2004, a change in the diagnosis and research of inflammatory demyelinating CNS diseases was evoked with the description of specific autoantibodies in patients with NMOSD (10). These antibodies are directed against aquaporin-4 (AQP-4), an abundant water channel in the CNS on astrocytic endfeets (11). However, a subgroup of clinically defined NMOSD patients are seronegative, and no marker is so far established for other differential diagnoses (12).

In animal models of MS (experimental autoimmune encephalomyelitis, EAE) a well-known target structure is myelin oligodendrocyte glycoprotein (MOG) (13), a protein comprising 245 amino acids that is exclusively expressed on the outermost surface of the myelin sheath and oligodendrocyte plasma membrane in the CNS, and which is easily accessible by a humoral immune reaction (14, 15). After passive immunization with tissue homogenates of CNS, the predominant antigen target in EAE is MOG, and inflammatory and demyelinating changes are enhanced by MOG antibodies (16, 17, 18, 19). Furthermore, in combination with complement, demyelination, and diseases relapses have been induced and MOG antibodies seem to be involved in macrophage mediated myelin destruction/phagocytosis (12).

Given these promising results, a multiplicity of studies have attempted to identify MOG antibodies in demyelinating CNS diseases. Numerous techniques and heterogeneous study populations have been included in these, leading to conflicting and inconsistent data on the prognostic and diagnostic value in MS (20–24). However, the establishment of methods similar to that used for the analysis of AQP-4 directed antibodies has enabled the reliable detection of antibodies against native correctly folded and glycosylated MOG (12, 25). With these cell-based assays, a humoral immune response against MOG has been consistently identified—initially in ADEM and subsequently in a subgroup of particular pediatric patients with inflammatory demyelinating CNS diseases (20, 22, 24, 26).

In the last few years, the MOG antibody-associated disorder spectrum has been rapidly broadening, making more data regarding clinical, radiological, and laboratory findings available, as well as elucidating immunopathogenesis. The current paper discusses the developing clinical spectrum, histopathological data, and immunopathogenesis, alongside the implications of the same for daily clinical practice.

## Clinical presentation and prognosis of MOG antibody-associated disorders

**Figure 1:**
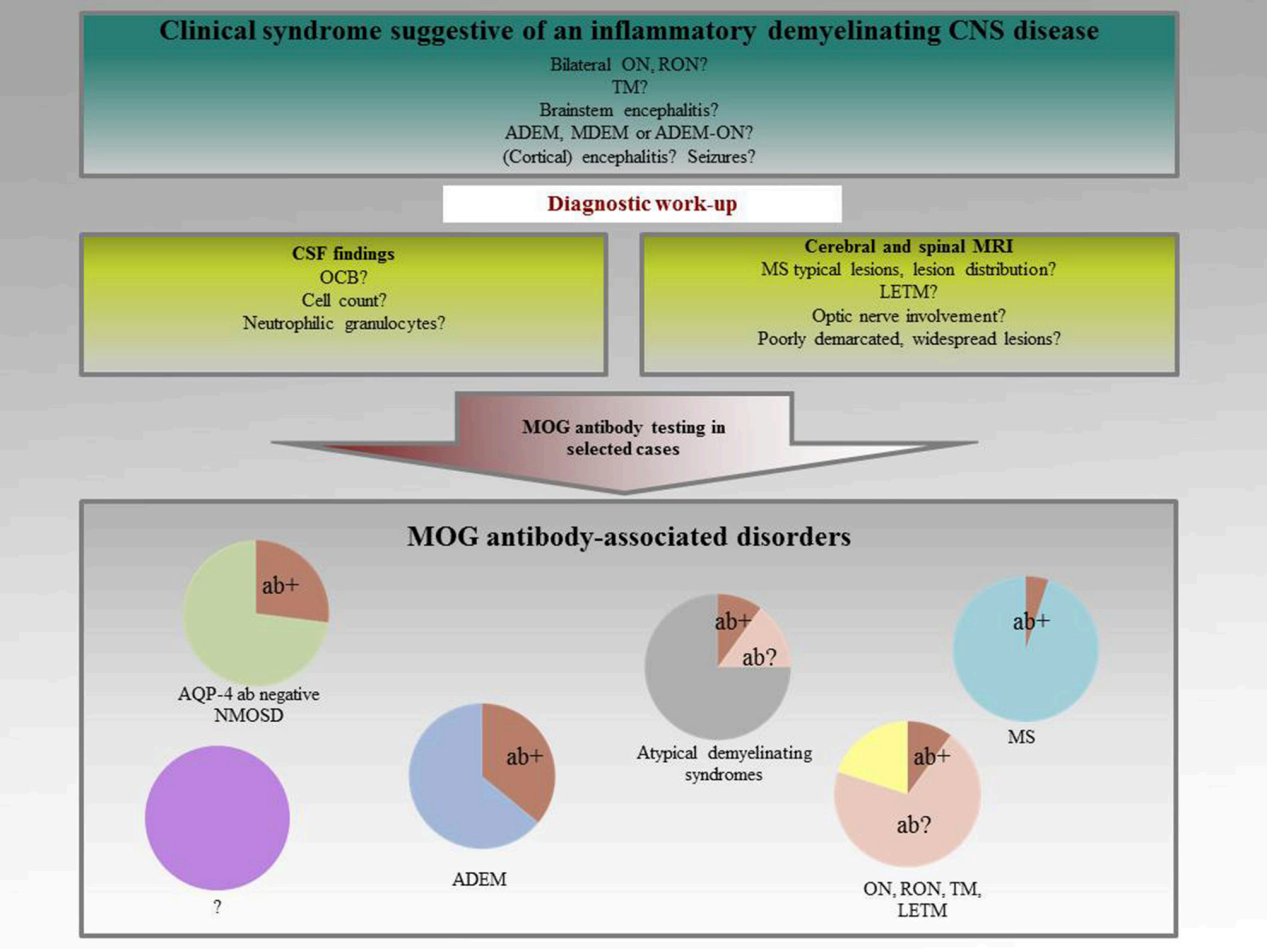
Spectrum of MOG antibody-associated disorders.

The first evidence for the potential use of antibodies against native MOG as a biological marker for ADEM was published by O'Connor et al. (26). Self-assembling radiolabelled MOG tetramers were established and a humoral immunoreactivity against MOG reliably identified in a subgroup of children with ADEM. Initially, these antibodies seemed to be associated with monophasic disease courses, predominantly present in children with an ADEM-like onset (27–29). Subsequent studies, however, revealed that the spectrum of MOG antibody-associated disorders is much broader. MOG antibodies have been found to be present in a subset of patients with ADEM, NMOSD, monophasic, and recurrent optic neuritis (ON), and transverse myelitis (TM), demyelinating syndromes overlapping with anti-NMDA receptor encephalitis or glycine receptor alpha 1 subunit antibody positive ON, Figure 1 (26, 27, 29–48). It is now well-accepted that MOG antibodies are in particular associated with ON and TM (49). In MS, a humoral immune response against MOG is only rarely seen (12). In atypical MS with a distinct clinical phenotype of e.g., severe brainstem and spinal cord involvement, immunoreactivity against MOG has been described in up to 5% of cases (50). In this subgroup, frequent relapses and insufficient responses to disease-modifying treatment seem to be a common feature. As co-incidence of MOG and AQP-4 immunoreactivity is an exception, disease mechanisms have been suggested to be at least partly different in these two entities (24).

However, clinical MOG antibody-positive patients can present with an NMOSD phenotype. Mader et al. were the first to describe the presence of MOG antibodies in this patient group (51). Subsequent studies supported the results: overall, in AQP-4 negative patients, MOG antibodies have a prevalence of 25% (12). In contrast to AQP-4 antibody-associated disorders with the well-defined clinical phenotype of NMOSD, in MOG antibody-associated disorders, the clinical presentation is less well-defined. Still, particularly in children, the sensitivity in ADEM is highest, at an average of 36% in different studies (12).

The two largest cohorts looking at the clinical features of MOG antibody-associated disorders were recently published (52, 49). Clinical presentation based on a trimodal distribution with age clusters of <20 years, 20–45 years, and >45 years, ADEM was most common in the age group <20 years; whereas ON (20–45 years) and bilateral ON (>45 years) were more frequent in adult patients with MOG antibody positive disorders (52). A short TM occurred in 14% of patients >45 years, but was rarely described in younger patients. The age-dependent clinical presentation was confirmed in a further study, with a predominance of ON found in adult onset MOG antibody-associated disorders, compared to a predominance of ADEM-like patterns in children as well as better recovery from neurological symptoms in children (53). The second largest study to include MOG antibody positive patients supported ON/TM as the main manifestations, given they represented clinical onset in over 90% of adult patients (49). However, NMOSD criteria (54) were fulfilled in only 19% of patients. Interestingly, in this study population, an encephalogenic phenotype was described with clinical signs of meningeal symptoms, retrograde amnesia, and seizures—uncommon symptoms in classical MS. Furthermore, seizures and encephalitis-like presentations are more common in MOG antibody-associated disorders compared to AQP-4 antibody positive diseases (55). Three recent case reports also found MOG antibodies to be associated with clinical presentation of cortical encephalitis and steroid responsiveness (56–58), indicating a new phenotype of MOG antibody-associated disorders.

Initial studies assumed MOG antibody-associated disorders to be monophasic, but it is now well-known that monophasic and recurrent diseases are both associated with MOG antibodies (59). In children, MOG antibodies are predictive not only of non-MS disease with a specificity of 100% but also of a recurrent non-MS disease course with a specificity of 75% including NMOSD, recurrent ON (RON), MDEM, and ADEM followed by optic neuritis (ADEMON) (48). Overall, 39% of MOG antibody positive children have been found to have a recurrent disease course, but only 5% a typical MS. It is important to note that as low levels of MOG antibodies were also measured in healthy and other neurological controls, a cut-off for positivity was in most studies defined as ≥1:160 (12). However, this study introduced a new cut-off for seropositivity, to increase the specificity for prediction of non-MS diseases with only a moderate decrease of sensitivity, at a titer of ≥1:1,280 (48).

Higher age, female sex, and MRI findings atypical of MS were found to be risk factors for a recurrent disease course. This reported risk was found to vary across different studies. Relapses were observed in 36% of 252 MOG antibody positive patients in the UK, with an annualized relapse rate of 0.2 (52), with the highest risk in patients with ON or NMOSD phenotypes. These relapse rates seem to be lower than those of AQP-4 antibody positive patients (37, 39, 49, 60). However, disease reoccurrence of up to 80% with an annualized relapse rate of 0.9 has been described as associated with a humoral immune reaction against MOG (61); in particular, a NMOSD phenotype seemed to be correlated with a relapsing disease (62). The highly variable data on further attacks and relapse rates may be due to the different characteristics of patients included for study, as well as the higher detection probability in relapsing diseases compared to monophasic diseases according to study design.

In several studies, a favorable outcome seemed to be associated with MOG antibodies (34, 39, 63). Patients seropositive for MOG antibodies less frequently suffer motor disability and have a better EDSS score after recovery compared to AQP-4 antibody positive patients (37). In patients with TM, the presence of MOG antibodies has also been associated with a better recovery from acute attack, indeed similar frequency of severe attacks at onset and similar relapse rates to AQP-4 antibody-associated disorders (64). Although, MOG antibody-associated ON is mainly a recurrent disease, accompanied by severe visual loss in the acute phase, visual recovery was found to be good (65); the outcome was better in MOG- compared to AQP-4 antibody positive patients correlating with a better preserved retinal fiber layer thickness (65, 66). However, in another study, severe functional loss was described in nearly half of MOG antibody positive patients and retinal axonal damage was similar in both conditions (61, 67). In a recent study, visual function outcomes and ambulation were significantly better in MOG antibody-associated disorders than in AQP-4 antibody-associated disorders; indeed, permanent disability was described in nearly half of the patients after a median disease duration of 16 months, and permanent bladder and erectile dysfunction in ~1 quarter of the MOG antibody positive patients (52). In a subgroup of adult MOG antibody positive patients, severe disease courses and lack of response to DMT were also noted (50). Though more data are necessary to confidently evaluate the prognostic value of MOG antibodies regarding disability, data indicates a favorable outcome at least in the majority of patients; however, severe disease courses with pronounced functional loss are possible, and may warrant early immunotherapy.

## Longitudinal analysis of MOG antibodies: implications for clinical practice

Prognostic assessment in MOG antibody positive patients who have had their first demyelinating event, with a possibility of an ensuing multiphasic disease course, is a challenge in clinical practice, and has important implications regarding further initiation of disease-modifying treatment. An association of longitudinal antibody level change with clinical course has been suggested (28, 29). Studies have also revealed an association of MOG antibody titer decrease with a monophasic disease course compared to stable or increasing titer in patients with multiphasic diseases (36, 45, 49, 50, 68). Persistent MOG antibodies have been predominantly found in recurrent non-MS diseases such as MDEM, NMOSD, and ADEMON (48). Furthermore, in a cohort of ON patients, 98% presented with persistent MOG antibodies, and of these 80% relapsed (65). A recent publication on adult and pediatric seropositive ADEM patients supported the clinical usefulness of serial MOG antibody testing for relapse prediction, as 88% with persistent MOG antibodies relapsed during long-term follow-up compared to 12% with transient antibodies (69). In the largest MOG antibody positive cohort to date, 72% of patients were persistently MOG antibody positive during the disease course; of these, 60% relapsed, whereas all transient antibody positive patients were relapse-free (52). Cobo-Calvo et al. confirmed the trend toward association of a relapsing disease with persistent antibodies only in a subgroup of patients; in some groups, no such association was observed (49). Similarly, Duignan et al. found persistent MOG antibodies in relapsing and monophasic diseases alike (70). In addition, one study showed that in adult MS patients, a subgroup had an immunoreactivity against MOG as well as associated severe brainstem and spinal cord involvement, frequent relapses, and a less favorable treatment response, with fluctuating and non-persistent antibody levels (50).

Promising results for the use of MOG antibodies as treatment biomarkers were published in 2017 in a study showing conversion to seronegativity during immune-directed therapies. The conversion was found to be a predictive marker for disease-free activity during the subsequent disease course (71). Although there is evidence for the potential use of serial testing as a long-term disease marker and potential treatment marker, more prospective data are necessary for the final evaluation of the predictive value of serial MOG antibody testing, as the results are in part inconsistent, and severe, relapsing disease courses have been described in patients with decreasing/disappearing antibody levels.

## Paraclinical findings and MOG antibodies

In ADEM, an intrathecal IgG synthesis as measured by IgG index or OCBs, is rare (72)—in contrast with MS, in which OCBs are present in over 90% of cases. OCBs are included in recent diagnostic MS criteria, and count for dissemination in time (1). Similar findings have been confirmed for MOG antibody-associated disorders: OCBs are uncommon, occurring in ~10% of cases, and cerebrospinal fluid (CSF) reactivity to MOG has only been shown in cases of high serum levels (27, 28, 46, 73). These findings indicate a peripheral production of MOG antibodies and secondary diffusion in the CNS similar to that in NMOSD (74). Possible explanations include: a direct CNS infection with leakage of CNS antigens in the periphery, and a secondary peripheral immune reaction against MOG (20); or a peripheral infection that stimulates MOG antibody production via molecular mimicry (20, 22).

Other routine CSF analyses were also comparable between MOG antibody-associated disorders and NMOSD. CSF pleocytosis was detected in 55–70% of cases, with neutrophilic granulocytes in more than half of patients and cell counts higher than in typical MS (37, 61, 75). In addition, similarities were found between CSF cytokine profiles in MOG antibody-associated disorders and AQP-4 antibody-associated NMOSD, with a predominant up-regulation of T helper 17 related cytokines in the latter, whereas in MS, T helper 1 related cytokines were found (75, 76), suggesting shared immunological pathomechanisms in the two diseases.

Besides clear differences in clinical and laboratory findings, MRI also provides a useful means of discriminating MOG antibody-associated disorders from other CNS demyelinating diseases, in particular MS. Brain MRI abnormalities at onset range from 40 to 77% (41, 49, 61, 77, 78) and supratentorial lesions have been found in nearly half of patients during the disease course and brainstem, respectively, cerebellar lesions in one third of the patients. Brain MRI abnormalities are associated with pathological CSF findings (49). According to the typical clinical manifestations of TM, the most common imaging finding is a longitudinally extensive transverse myelitis (LETM) or a short TM (61). In MOG antibody-associated ON, typical imaging characteristics are a contrast enhancement of the optic nerve, a perineural enhancement in a proportion of the patients, and in 80%, more than half of the pre-chiasmic optic nerve length being affected (79, 65). Lesion distribution in children seems to be age-dependent, with poorly demarcated, widespread lesions in younger children, in contrast with a normal brain MRI in older children (80). It has been possible to distinguish MOG antibody-associated NMOSD from MS with a specificity of 95% and a sensitivity of 91% by employing predefined MRI criteria for lesion distribution, including Dawson's fingers, subcortical *U* fiber lesions, and lesions adjacent to the lateral ventricles, as typical for MS (81). A subsequent study confirmed these results, and was able to accurately discriminate MS from MOG antibody-associated disorders by the presence of ovoid lesions adjacent to the body of the lateral ventricles, Dawson's fingers, and T1 hypointense lesions, whereas fluffy lesions and three lesions or less were typical for MOG antibody-associated disorders (82). However, there was an overlap between MRI characteristics for AQP-4 and MOG antibody-associated disorders. Moreover, a further study could not identify typical radiological features to discriminate between the diseases; indeed, thalamus, and pons lesions were more common in MOG antibody-associated disorders, and in 16% of patients, a cortical involvement, and in 6% a leptomeningeal enhancement, was described (49).

Although MRI is variable in MOG antibody-associated disorders, depending on the clinical presentation and age of the patient, it is an important diagnostic tool. In the absence of an unique imaging finding, typical features of MOG antibody positive patients are characterized as a normal brain MRI or large, confluent, poorly marginated MRI lesions (if clinically presenting with ADEM), LETM, perineural enhancement of the optic nerve, brainstem and hypothalamic lesions, and a leukodystrophy-like MRI pattern (25, 83).

## Diagnostic recommendations for MOG antibody-associated disorders

The International Panel on Diagnosis of Multiple Sclerosis published in 2017 its most recent diagnostic criteria for MS. The revised diagnostic criteria were based on further knowledge of a combination of clinical, MRI, and CSF findings, and emphasized the important role of OCBs in the diagnosis of MS and in reducing the risk of misdiagnosis (1). Although MOG antibody testing was not included in the revised criteria, due to a lack of full validation of antibody testing, special clinical situations were described for which antibody testing was recommended.

NMOSD and MS are often precisely discriminated by clinical and paraclinical features (84), of which the important therapeutic consequences regarding DMT requires special attention. NMOSD, therefore, should be considered in every suspected case of MS (1). The presence of antibodies against MOG and AQP-4 should be tested for in patients with clinical symptoms suggestive of NMOSD, such as bilateral ON, severe brainstem involvement, or LETM, in special patient groups with a high risk of NMOSD, if there is evidence of large cerebral lesions, if MS criteria of dissemination in space are not fulfilled, or if brain MRI is normal (1). As lack of OCBs is a very rare finding in typical MS; MOG antibody testing should be considered in OCB negative MS patients. In pediatric onset MS, antibody testing can support the diagnosis of AQP-4 negative NMOSD, ADEM followed by RON or with including chronic relapsing inflammatory optic neuropathy (CRION). In 2017, Hacohen et al. more precisely described the routine diagnostic use of MOG antibody testing for pediatric patients in clinical practice, and proposed a diagnostic algorithm for any episode of CNS demyelination (83). According to the revised McDonald criteria, first of all the diagnosis of MS should be evaluated by spinal and brain MRI and CSF findings. Given the 2010 McDonald diagnostic criteria (85), in this cohort diagnosis MS is also reliable in children independent of age and no further diagnostic steps are required. However, if there are features of NMOSD or ADEM in cases where the patient is AQP-4 antibody negative, MOG antibody analysis is strongly recommended. In contrast, preceding criteria advised to apply the McDonald criteria with caution for children under 12 years, as the validation of the predictive value is lacking (86). In addition, MOG antibodies are much more frequent in children; therefore, less stringent indications for antibody testing should be implemented in the clinical practice.

Other red flags indicating the usefulness of MOG antibody testing identified in the study of Hacohen et al. included poorly marginated lesions located in the cerebellar peduncle and a leukodystrophy-like MRI pattern. As MOG antibody positive patients have distinct clinical features (being young, less frequent area postrema syndrome, typically presenting ADEM initially, lower disability during follow-up, a longer time interval till the first relapse), the authors regard MOG antibody-associated disorder as a new phenotype, discriminating it in terms of its diagnostic algorithm from MS, AQP-4 antibody positive NMOSD, and antibody-negative recurrent demyelinating syndrome (83).

Due to the rising relevance, in 2018 an international recommendation based on expert consensus was proposed for indication of antibody testing for patients with a demyelinating CNS disease of suspected autoimmune etiology and either a monophasic or relapsing disease course (25). Jarius et al. proposed the urgent necessity of stringent indications for MOG antibody testing, as screening for a rare biomarker in large, unselected patient cohorts significantly decreases the predictive power of a test (25). This limitation applies particularly to adult patients, as in children MOG antibodies are more common. Based on a combination of clinical, imaging, and laboratory findings, MOG antibody testing should be performed in patients with high risk of a MOG antibody-associated disorder and/or in the case of findings that are atypical for MS. Concrete antibody testing indications are: “Monophasic or relapsing acute ON, myelitis, brainstem encephalitis, encephalitis, or any combination thereof, AND radiological or, only in patients with a history of ON, electrophysiological (VEP) findings compatible with CNS demyelination” (25). In addition, at least one further finding is necessary, of clearly defined MRI, Fundoscopy, CSF, or clinical features, or typical treatment response. Among others, a progressive disease course, progressive lesion load shown by MRI during clinically inactive time periods, AQP-4 AND MOG antibody positivity, and MOG IgM antibodies are regarded as red flags for a false positive result.

As discussed above, no exact clinically unique phenotype has been identified in patients with MOG antibodies. However, MOG antibody-associated disorders share similar features and a common treatment response, making their inclusion in diagnostic criteria for all patient age ranges of important clinical relevance. Therefore, two research groups independently suggested diagnostic criteria for MOG antibody-associated disorders, the newly introduced entity was termed as “MOG encephalomyelitis” (25), respectively, “MOG IgG associated disorders” (69). Jarius et al. propose the possible diagnostic criteria for “MOG encephalomyelitis” in adult patients, as including MOG antibody seropositive patients with either a monophasic or relapsing ON, TM, brainstem encephalitis, or encephalitis (or a combination of these syndromes), if MRI or electrophysiological findings are compatible with CNS demyelination (25). In the second proposal for diagnostic criteria of Lopez-Chiriboga et al., similar findings are required: MOG-IgG seropositivity measured by a cell-based assay with clinical findings of ADEM, ON, CRION, TM, brain or brainstem syndrome compatible with demyelination, or any combination of the described syndromes, after exclusion of other differential diagnoses (69).

These suggested criteria are preliminary: validation experiments are essential for confirming final use in clinical practice. Furthermore, depending on future data and antibody testing methods, which may offer improvements in sensitivity and specificity, adaptions will be necessary. In particular in large, experienced MS centers, screening for MOG antibody positivity in typical MS cohorts, and critical consideration of results, could yield enhanced knowledge of the whole spectrum of MOG antibody positive disorders.

## How to test for MOG antibodies

Several detection methods have been applied in identifying MOG antibodies in inflammatory demyelinating CNS diseases. Given the inconsistent results generated by ELISA and immunoblot in MS patients, these techniques are now regarded as obsolete (21). However, reliable results have been recorded with cell-based assays expressed in human cells using immunofluorescence or fluorescence-activated cell sorting. With this method, the expression of natural conformation full length native MOG at the cell surface is possible, and subsequently, so is the detection of antibodies targeting human MOG. Different expression vectors, cell lines, and read-out systems have been reliably used. Immunohistochemistry is not recommended, due to reduced sensitivity depending on the tissue donor, and limited data regarding specificity (12, 87). As mentioned above, a cut-off is important, as in healthy individuals as well as other neurological controls, low-titer antibodies are detectable, leading to a lack of disease specificity for low-titer MOG antibodies. Most studies have used a cut-off of ≥1:160 (88). A higher prognostic specificity has been described using a higher cut-off titer for positivity, but further prospective studies are required for the evaluation of optimal cut-off.

Waters et al. were able to improve the test using an IgGI-specific secondary antibody, in response to the problem of cross-reactivity of the anti-human IgG secondary antibody with IgM and IgA antibodies. This optimization increased the specificity of the MOG antibody assay in cases of non-MS disease, and the method provided class II evidence for the discrimination of non-MS CNS demyelinating disorders from MS (89). As an alternative, IgG Fc antibodies can be used (25). As data suggests peripheral production of MOG antibodies, analysis of serum samples results in higher specificity than CSF samples (28, 46), and CSF analysis is only recommended in rare cases (46).

Although the value of longitudinal testing warrants further evaluation, the current authors recommend serial analysis of positive patients at 6–12 months. As there is no evidence for seroconversion from negative to positive (48), there is no expectation of additional information by retesting negative patients during the disease course. However, as there is no gold standard for MOG antibody analysis, in particular cases with clinical and paraclinical findings suggestive for a MOG antibody associated disorder retesting is reasonable.

## Lessons from neuropathology

Only limited data, mainly by single case reports, are available regarding MOG antibody positive patients and underlying histopathology. However, the existent neuropathological findings are consistent and show in most cases MS pattern II pathology (12). To the current authors' knowledge, to date only nine cases with available neuropathology are described in the literature, Table 1 (47, 56, 58, 90–94). Most cases revealed MS pattern II lesions with demyelination, relatively preserved axons, pre-oligodendrocytes, an absence of myelin, and myelin-laden macrophages. The inflammatory hallmark is an infiltrate consisting of T cells as well as a complement and antibodies (12)—indicative of humoral pathogenesis in these cases. The clinical presentation of MOG antibody positive patients with MS pattern II pathology varies, and includes cases with CIS, MS, NMOSD, recurrent LETM followed by tumefactive lesions, and atypical inflammatory demyelinating CNS syndromes (47, 58, 90–93, 94). Tough the clinical presentation corresponded to NMOSD, the typical pathological hallmarks of NMOSD with AQP-4 and astrocyte loss, necrosis, complement activation, focal perivascular or confluent extensive demyelination, eosinophilic, and neutrophilic cell infiltration (95), and thickened hyalinized vessel walls (96) were missing.

**Table 1:** Histopathological findings in MOG antibody-associated disorders. Modified after (12, 24).

**Sex**	**Age**	**Clinical diagnosis**	**Neuropathological classification**	**Inflammatory infiltrates and confluent demyelination^a^**	**Perivascular complement deposition and/or AQP-4 loss^b^**	**Eosinophilic cell infiltration^b^**	**Comment**	**References**
M	71	Fulminant encephalomyelitis	MS pattern II, in addition lesions with complement activation and AQP4 loss	+	+ in a single lesion of optic chiasm	nr	Late seroconversion to low-titer AQP-4 antibody positivity during disease course	(91)
M	46	Encephalitis, ADEM-like lesions and unilateral optic neuritis	Mild inflammatory changes	na	na	na	No demyelinating lesions	(56)
F	29	Cerebral cortical encephalitis with epilepsy and bilateral optic neuritis	Inflammatory infiltrates in the cortex and subcortex	na	na	na	No demyelinating lesions	(58)
F	63	CIS	MS pattern II	+	–	–	–	(93)
W	49	RRMS	MS pattern II	+	–	–	–	(90)
M	49	ADEM	MS pattern II with an overlap of MS pattern III	+	–	–	Oligodendrocyte apoptosis and loss	(94)
M	34	ADEM	MS pattern II	+	–	–	–
F	66	Recurrent myelitis and brainstem involvement followed by tumefactive bilateral lesions	MS pattern II	+	–	–	–	(92)
F	67	NMOSD, recurrent myelitis followed by cerebral tumefactive lesions	No pattern classification, inflammatory demyelination without astrocyte loss	+	nr	nr	–	(47)

a *Typical neuropathological findings of MS and NMOSD*.

b *Typical neuropathological findings of NMOSD, nr, not reported; na, not applicable*.

Similar results have been obtained for ADEM. No clinical ADEM MOG antibody positive case to date, according to the diagnostic criteria, has the ADEM typical neuropathological findings with perivenous demyelination (compared to confluent demyelination in MS) (97) and cortical microglial activation (35). In accordance with this, it has previously been shown that 9% of patients with ADEM according to clinical criteria were misdiagnosed, since the pathology was MS typical and the patients developed MS during long-term follow-up (97). Complications in ADEM diagnosis are still possible with biopsy, as an overlap of confluent and perivenous demyelination has been described. However, two recently published MOG antibody positive cases presented with a distinct pathology and clinical presentation (56, 58). In the first case, a bilateral cortical frontal steroid-responsive encephalitis with ADEM-like lesions and ON was associated with MOG antibodies and mild inflammatory changes with intact myelin sheaths (56). Comparably, in the second case, of cerebral cortical encephalomyelitis, epilepsy, and steroid responsiveness, biopsy revealed slight inflammation without distinct demyelination, and in contrast to the first case, mild loss of MOG (58). Whether these two cases extended the spectrum of MOG antibody-associated disorders to a subgroup of cortical inflammatory encephalitis without pronounced demyelination needs to be further elucidated.

To summarize the available rare data, MOG antibody-associated disorders seem to be mainly associated with MS pattern II pathology, independent of clinical features, pointing to a distinct humoral-mediated disease group of demyelinating CNS diseases.

## Evidence for the pathogenic role of MOG antibodies

In animal models, it is well-established that MOG antibodies have a pathogenic effect (12); however, in humans, the role of MOG antibodies in disease pathogenesis is less clear and still under debate, including the subjects of direct, antibody-mediated cell induced tissue destruction or their presence of a bystander phenomenon.

Initial studies showing evidence for a pathogenic effect of humoral immune response against MOG involved purified human MOG antibodies; these antibodies were able to induce cell death of MOG-expressing cells, as well as natural killer-cell mediated cell death, with the extent of cell damage dependent on antibody levels (27, 30). In addition, MOG antibodies belong mainly to the complement binding IgG1 subtype, and have been found to be able to activate the complement cascade, finally leading to complement-dependent destruction of MOG expressing cells (31, 51). A disruption of the oligodendrocyte cytoskeleton, with the effect of a functional modification, has been described (73); however, results from *in vivo* studies of the ability of MOG antibodies to damage tissue are inconclusive. Patient purified antibodies injected in EAE increased demyelination (30), and reversibly damaged axons, though no inflammatory reaction or complement deposition was induced (98). One possible explanation is that human and rodent MOG differs, and human MOG antibodies do not recognize rodent MOG (99).

However, in CNS antigen-presenting cells (APCs), accumulation of MOG antibodies has been described, with a subsequent activation of autoreactive T cells, as well as a MOG antibody induced Fc-mediated APC recognition of MOG, followed by induction of peripheral autoreactive T cells (100, 101). A recent study confirmed the pathogenic effect on rodents of affinity-purified MOG antibodies transferred from patients. These purified MOG antibodies were not only able to mediate MS pattern II pathology with typical immunoglobulin-mediated tissue destruction, but also induce, in combination with MOG reactive T-cells, a clinical disease with enhanced T-cell recruitment and reaction (102). Importantly, results revealed that MOG antibodies alone did not induce inflammation and tissue destruction—their interdependence with T-cells was required to evolve their pathogenic potential.

Evidence is arising that MOG antibodies have a pathogenic potential, but the exact pathomechanism and the synergy with T cells requires further elucidation. However, if MOG antibodies are mainly bystanders and only slightly contribute to disease development and pathogenesis, their important role as disease biomarkers is obvious.

## Treatment

As there have been no controlled treatment trials in MOG antibody positive diseases, therapy regimes are based on the suspected individual prognosis and clinical experiences. In an acute attack, similar approaches are used as in other inflammatory demyelinating CNS diseases such as MS (intravenous methylprednisolone and plasma exchange). A favorable recovery has been demonstrated in 70–90% of patients given intravenous methylprednisolone (39, 103). Long-term treatment with corticosteroids reduces the risk of relapse and cessation has been associated with breakthrough disease (103). Jarius et al. describe similar results, with a full recovery in 50% of cases, partial recovery in 44%, and no recovery in 6% (61). Of particular importance is that tapering or finishing of corticosteroids was followed by a flare-up of the disease and early relapses (25, 52, 103). Thus, some authors have favored long-term steroid treatment over 6 months, given alongside other immunomodulatory or immunosuppressive drugs. When there is suggestion of an antibody-mediated immune reaction, plasma exchange, which is normally initiated if corticosteroid therapy is insufficient, is promising for managing the acute attack. Use of plasma exchange seems to be associated in several reports with a better outcome and improved neurological deficits after the failure of corticosteroids (50, 80). However, though plasma exchange seems mainly to be followed by a good functional outcome, in a substantial proportion of patients, only partial recovery was achieved (61). On balance, plasma exchange seems to be a reasonable therapy after the treatment failure of corticosteroids, or in selected patients as an early treatment option.

A challenge in MOG antibody-associated disorders is the choice of long-term immunotherapy, since clinical courses and prognoses substantially vary between individuals. When considering the underlying pathogenesis as involving B cells and antibodies, therapies directing the humoral immune response may prove most promising.

Although the data shows that recurrent disabling disease courses are common with MOG antibody-associated disorders, they are treated less often than AQP-4 associated diseases (104). Only 40% received a long-term maintenance therapy (52). As mentioned above, a combination of corticosteroids and other immune-mediated therapies seems favorable. Ramanathan et al. found a reduction of relapse rates with different immunotherapies such as azathioprine, rituximab, and mycophenolate, maintenance corticosteroids and rituximab being most effective in preventing disease activity (103). In addition, further studies confirmed the positive effect of immunosuppression/immunomodulation, including azathioprine, methotrexate, and rituximab, on the risk of relapse and the annualized relapse rate (23, 52, 49), in particular if treatment is maintained for more than 3 months (52). Recently, a study including children with relapsing MOG antibody associated disorders demonstrated a benefit of intravenous immunoglobulins on the annualized relapse rate (78). Classical MS drugs such as natalizumab, interferon, and glatirameracetat showed no treatment efficacy (61). To the knowledge of the current authors, so far only one case has been published detailing treatment with alemtuzumab, a highly effective treatment in MS. Similar to reports of alemtuzumab use in AQP-4 antibody NMOSD (105), treatment failed and disease activity resumed (106). The failure of a treatment effective in MS is well-known in NMOSD, suggesting a distinct pathomechanism in antibody-associated disorders. In conclusion, promising treatment regimes include maintenance corticosteroids and rituximab, although for a more definitive statement, prospective controlled trials are required.

## Conclusion

Inflammatory demyelinating CNS diseases include a broad spectrum of different diseases, among which single diseases might show distinct clinical phenotypes and prognoses. For disease stratification, prognostic evaluation, treatment decisions, and patient counseling an early diagnosis is important. Diagnostic procedures now include a combination of clinical, imaging, and laboratory findings. However, correct diagnosis at disease onset is still a challenge and an exact prognostic estimation regarding occurrence of relapses and disability is remains out of reach. Biomarker research has therefore been a focus of interest for several decades, given that in MS, in particular, new treatment allows for early therapy initiation. However, since newly available treatments are not only more effective but also more aggressive, carrying more side effects and risks, overtreatment should be avoided.

Over the past few years our knowledge of clinical, imaging, and laboratory data regarding MOG antibody-associated disorders has evolved. Clear differences in this spectrum have not only been found with MS, but also, to a lesser degree, with AQP-4 associated disorders. Although there is no unique clinical phenotype, clinical presentation, prognosis, and treatment response is distinct in this demyelinating CNS disease subgroup. In particular, in MOG antibody-associated NMOSD a different immunopathogenesis, with an oligodendrogliopathy rather than a classical astrocytopathy, is suggested. These differences are mirrored in the histopathological findings of MOG antibody-associated disorders, where there is a preponderance of MS pattern II findings. This finding is clearly different from AQP-4 associated disorders, and suggests that other therapeutic strategies might be promising. An integration of the two diseases would be short-sighted, as there are not only important implications for further research but also for patient counseling and treatment considerations.

Various research groups have published diagnostic recommendations for MOG antibody-associated disorders and introduced them as a new spectrum disorder. International cooperation for the development of diagnostic consensus criteria, either as stand-alone or for inclusion in the MS or NMOSD criteria, would constitute further important progress. In addition, serial testing is now upcoming in the generation of prognostic, and perhaps also therapeutic, biomarkers; its routine use in clinical practice warrants further prospective trials, in particular for patients undergoing long-term treatment. Overall, MOG antibody-associated disorders should, it is suggested, be classified as distinct spectrum disorders, though research is still in its early stages in understanding the exact underlying pathomechanism and its prognostic implications.

## Author contributions

All authors listed have made a substantial, direct and intellectual contribution to the work, and approved it for publication.

### Conflict of interest statement

The authors declare that the research was conducted in the absence of any commercial or financial relationships that could be construed as a potential conflict of interest.

## Abbreviations

ADEM: acute disseminated encephalomyelitis
ADEMON: acute disseminated encephalomyelitis followed by optic neuritis
AQP-4: aquaporin-4
CSF: cerebrospinal fluid
CRION: chronic relapsing inflammatory optic neuropathy
EAE: experimental autoimmune encephalomyelitis
LETM: longitudinally extensive transverse myelitis
MDEM: multiphasic disseminated encephalomyelitis
MS: multiple sclerosis
MOG: myelin oligodendrocyte glycoprotein
NMOSD: neuromyelitis optica spectrum disorder
OCBs: oligoclonal bands
ON: optic neuritis
RON: recurrent optic neuritis
TM: transverse myelitis.
